# Carnosic acid slows photoreceptor degeneration in the *Pde6b*^*rd10*^ mouse model of retinitis pigmentosa

**DOI:** 10.1038/srep22632

**Published:** 2016-03-10

**Authors:** Kai Kang, Matthew J. Tarchick, Xiaoshan Yu, Craig Beight, Ping Bu, Minzhong Yu

**Affiliations:** 1 Department of Ophthalmic Research, Cole Eye Institute, Cleveland Clinic Foundation, Cleveland OH, USA; 2 Louis Stokes Cleveland Veterans Affairs Medical Center, Cleveland, OH, USA; 3 Department of Ophthalmology, Loyola University Chicago, Maywood, IL, USA; 4 Department of Ophthalmology, Cleveland Clinic Lerner College of Medicine of Case Western Reserve University, Cleveland OH, USA

## Abstract

The photoreceptor cell death associated with the various genetic forms of retinitis pigmentosa (RP) is currently untreatable and leads to partial or complete vision loss. Carnosic acid (CA) upregulates endogenous antioxidant enzymes and has proven neuroprotective in studies of neurodegenerative models affecting the brain. In this study, we examined the potential effect of CA on photoreceptor death in the *Pde6b*^*rd10*^ mouse model of RP. Our data shows that CA provided morphological and functional preservation of photoreceptors. CA appears to exert its neuroprotective effects through inhibition of oxidative stress and endoplasmic reticulum stress.

Retinitis pigmentosa (RP) is a class of inherited diseases which are characterized by the gradual degeneration of rod photoreceptors followed by cone photoreceptor cell dysfunction and death^1^. RP is a significant cause of vision loss, and affects approximately 1 in 3,700 people^2^. The initial symptoms of RP impact the peripheral retina. RP in late stages will involve central vision and may result in legal blindness^3^. Although effective treatments for RP should start early in life, there are currently no effective medications available for controlling the development of RP due to the limited therapeutic benefits or potential side effects of currently available treatment options^4^,^5^. It is therefore important to search for novel therapeutics for RP treatment^6^.

Photoreceptors work in a very challenging environment characterized by high oxygen supply^7^, excessive light exposure^8^, dim ambient light and active metabolism^9^. These stressors induce oxidative damage of the biological macromolecules that comprise photoreceptors^10^. Increasing evidence obtained from animal models of RP suggests that oxidative stress^11^,^12^, as well as endoplasmic reticulum (ER) stress^13^, may be the critical mechanisms underlying photoreceptor damage and death^14^,^15^,^16^. Consistent with this hypothesis, a number of studies have demonstrated that early administration of agents that inhibit oxidative stress could significantly decrease the rate of photoreceptor cell death in animal models of RP^11^,^17^,^18^.

Carnosic acid (CA) is a potent antioxidant isolated from *Rosmarinus officinalis*. CA can readily cross the blood-brain barrier^19^ and exert its protective effects after conversion from its catechol form to an electrophilic quinone form. This conversion allows CA to bind to Kelch-like ECH-associated protein 1 (Keap1) in the cytoplasm and subsequently release protective transcription factors^20^,^21^. Unlike other antioxidants, CA does not deplete the endogenous antioxidant glutathione^20^.

The *Pde6b*^*rd10*^ (*rd10*) mouse is a well-characterized model of RP^22^,^23^. The *rd10* mouse carries a missense mutation in exon 13 of the beta subunit of the rod phosphodiesterase gene (*Pde6b*)^22^,^23^, mutations in which also cause human RP^24^,^25^. In *rd10* mice, rod cell death begins around postnatal day (P) 18^26^, and is near complete by P35^27^. In this study, we demonstrate that CA slows rod degeneration in the *rd10* mouse, by reducing oxidative stress and ER stress.

## Results

### Electroretinography

**Figure 1:**
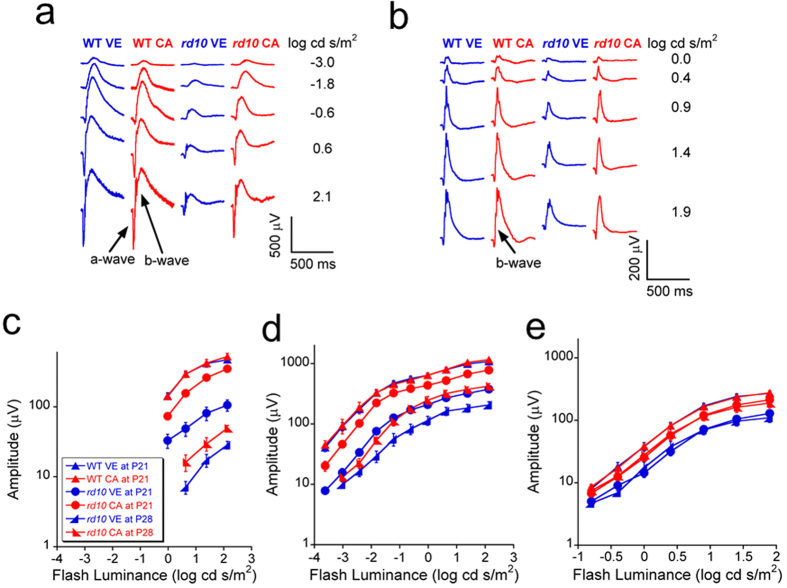
ERG results obtained from *rd10* mice treated with vehicle (n = 12 at P21; n = 5 at P28) and CA (*n* = 10 at P21; *n* = 8 at P28), and from wild-type (WT) mice treated with vehicle (*n* = 4 at P21) and CA (*n* = 4 at P21). (**a**) Typical dark-adapted ERG waveforms at P21. (**b**) Typical light-adapted ERG waveforms at P21. (**c**) Luminance-response curves of dark-adapted ERG a-wave at P21 and P28. (**d**) Luminance-response curves of dark-adapted ERG b-wave at P21 and P28. (**e**) Luminance-response curves of light-adapted ERG b-wave at P21 and P28. The error bars indicate standard errors. Under both dark-adapted and light-adapted conditions, the ERG a- and b-wave amplitudes were significantly higher in CA treated group than in the vehicle group at P21 (all *p* < 0.01) and P28 (all *p* < 0.05), while the difference was not significant in the WT groups (all *p* > 0.05).

Electroretinography (ERG) was used to compare outer retinal function of mice (Fig. 1). Figure 1a,b compare representative ERG recordings made from wild-type (WT) and *rd10* mice at post natal day (P) 21 under dark-adapted and light-adapted conditions, while Fig. 1c–e present summary luminance-response functions obtained across all animals studied at P21 and P28. Consistent with prior characterization of the *rd10* retina^28^, the amplitude of the ERGs obtained from vehicle treated *rd10* mice were markedly reduced in comparison to WT in both dark-adapted (1a,c,d) and light-adapted (1b,e) stimulus conditions at P21 and P28. CA treatment did not impact ERG amplitude in WT mice (all *p* > 0.05). In comparison, responses of *rd10* mice treated with CA were much larger than those given vehicle at P21 (all *p* < 0.01) and P28 (all *p* < 0.05), respectively. Significant (all *p* < 0.05) improvement at P21 and P28 was noted for the a-wave, generated by rod photoreceptors, and the b-wave, which reflects rod (1d) or cone (1e) depolarizing bipolar cells. These results indicate that CA can delay the degeneration of rod and cone photoreceptors in *rd10* mouse retina. While both CA and vehicle treated groups showed significantly lower ERG a- and b-wave amplitudes at P28 than at P21 in dark-adapted condition (a-wave: all *p* < 0.01; b-wave: all *p* < 0.05), the decrease of ERG b-wave amplitude was not significant in light-adapted condition (all *p* > 0.05). It is consistent with the finding that the extent of ERG amplitude reduction is more severe in dark-adapted condition compared to light-adapted condition at specific age in *rd10* mice^29^.

### Detection of cell death by terminal deoxynucleotidyl transferase dUTP nick end labeling

**Figure 2:**
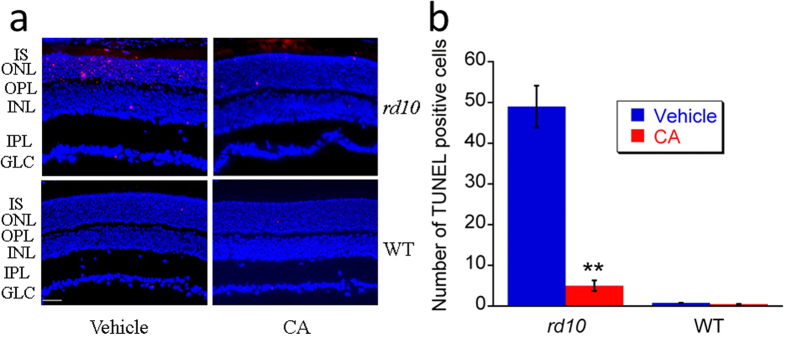
CA treatment reduced retinal cell death in *rd10* mice at P21. (**a**) Representative images (20×) of TUNEL assay in *rd10* mice and WT mice treated with vehicle or CA. The red spots indicate the TUNEL-positive cells. Scale bar indicates 50 μm. (**b**) Average number of dead cells in retinas of *rd10* mice and WT mice. Data was expressed as mean ± SD (*n* = 3). **p < 0.01 *vs*. vehicle group.

To better understand the impact of CA on the *rd10* retina, we stained retinal sections at P21 using a terminal deoxynucleotidyl transferase dUTP nick end labeling (TUNEL) assay^30^,^31^,^32^. In the representative images shown in Fig. 2, TUNEL-positive cells appear as red spots. In the WT retina, TUNEL-positive cells were seen only rarely. In *rd10* retinas, TUNEL-positive cells were mainly seen in the outer nuclear layer (ONL) and their frequency was significantly reduced by treatment with CA (*p* < 0.01). These TUNEL-positive cells may include apoptotic cells^33^ and all kinds of dying cells^30^.

### Histological analysis

**Figure 3:**
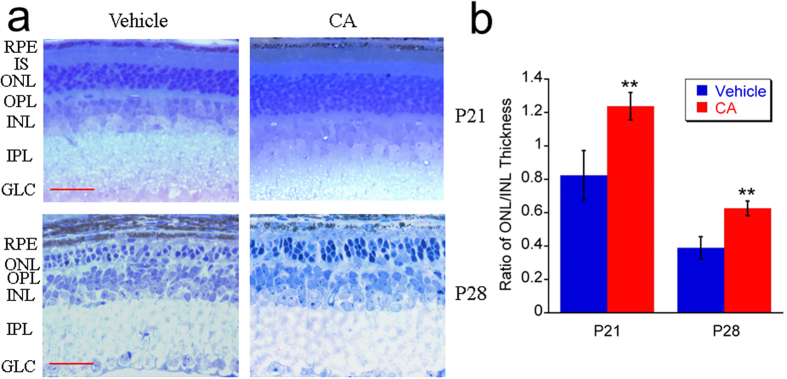
CA treatment ameliorated the reduction of ONL thickness in *rd10* mice. (**a**) Representative images (40×) of retinal histology from *rd10* mice treated with vehicle or CA. Scale bar indicates 30  μm. (**b**) Ratio of ONL to INL thickness. Data was expressed as mean ± SD in vehicle group (*n* = 4 at P21, *n* = 6 at P28) and CA group (*n* = 6 at P21 and *n* = 6 at P28). **p < 0.01 *vs*. vehicle group.

Figure 3 shows the representative images of histology of *rd10* mice at P21 and P28. Because about 97% of photoreceptor nuclei in the ONL of mouse retina are rod cells^34^, the thickness ratio of ONL to inner nuclear layer (INL) was used as an index to assess the rod death. The reduction of the ONL thickness was significantly ameliorated after the treatment with CA in *rd10* mice at P21 and P28 (all *p* < 0.01), while the ratio of ONL to INL is lower at P28 compared to P21 in vehicle treated mice as well as in CA-treated mice. These data prove the neuroprotective effect of CA against the degeneration of photoreceptors in this mouse model and confirms that the ONL degeneration develops with age.

### Analysis of protein pathway biomarkers

#### Upregulation of Nrf2 expression

**Figure 4:**
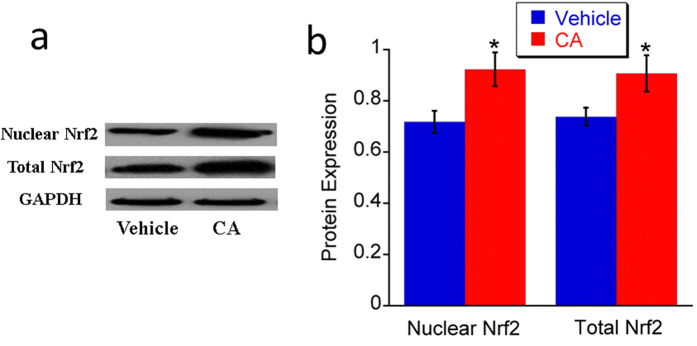
CA treatment enhanced Nrf2 expression in *rd10* retina at P21. **a**. Representative images of western blot for total Nrf2 and nuclear Nrf2. **b**. Average protein expression of total Nrf2 and nuclear Nrf2. *n* = 3 in each group. The error bars indicate standard deviations. *p < 0.05 *vs*. vehicle group.

Nuclear factor (erythroid-derived 2)-like 2 (Nrf2) is a transcription factor that can respond to increased level of reactive oxygen species and promote the expression of phase II enzymes and endogenous antioxidants to restore the homeostasis of reactive oxygen species. Nrf2 has been recognized as a transcription factor that involves mechanisms of cellular defense response against oxidative stress. Nrf2 protein stays in the cytoplasm by binding to Keap1 and remains inoperative^35^. Upon activation, activators interact with a cysteine on Keap1 to release Nrf2 and resultantly Nrf2 moves into the cell nucleus, binds with antioxidant response element, and induces expression of cytoprotective target proteins, including phase II detoxifying enzymes, antioxidant proteins, and the molecular proteasome/chaperones^36^,^37^. Because CA was reported to play its role of antioxidant by activating Nrf2 pathway^20^, the expression of Nrf2 was determined by western blot in this study. The antibody only tested the Nrf2 protein which disassociated from Keap1 and relocated to nuclei. Our data shows that both total and nuclear Nrf2 protein expression was increased in the retina of the CA treated *rd10* mice (Fig. 4), which confirms that CA treatment can activate Nrf2 pathway.

#### Downregulation of expression of p-JNK and p-p38

**Figure 5:**
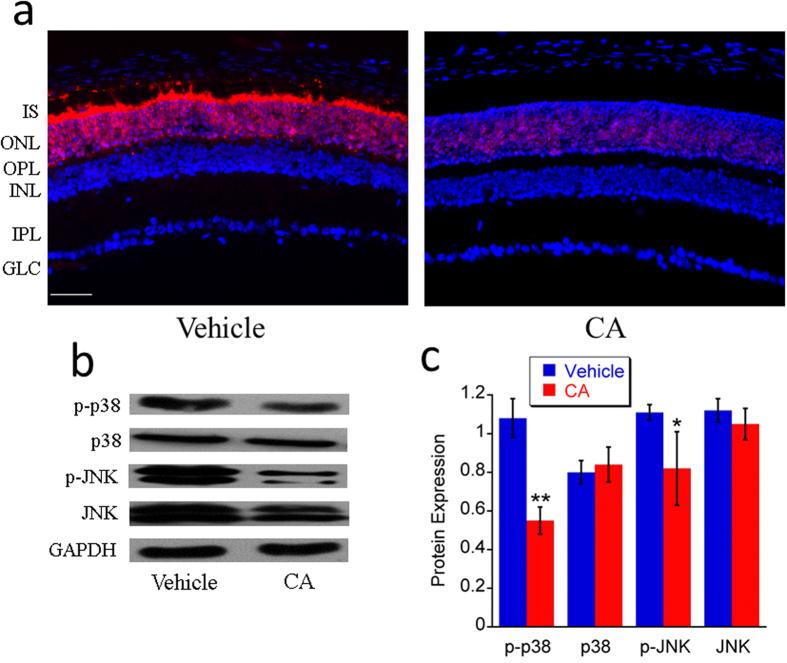
CA treatment downregulated the expression of some protein markers of oxidative stress in *rd10* retina at P21. **a**. Representative images (20x) of retinal immunohistochemistry for p-JNK (red). Scale bar indicates 50 µm. **b**. Representative images of western blot of JNK, p-JNK, p38 and p-p38. **c**. Average protein expression of JNK, p-JNK, p38 and p-p38. *n* = 3 in each group. The error bars indicate standard deviations. *p < 0.05, **p < 0.01 *vs*. vehicle group.

There are three well-defined subgroups of MAPKs: the extracellular signal regulated kinases (ERKs), the c-Jun N-terminal kinases (JNKs), and p38 MAPKs (mitogen-activated protein kinases). Each of these subgroups is activated by a cascade of phosphorylation events and involved in the regulation of gene expression, differentiation, cell survival and death^38^. JNK and p38, subgroups of MAPKs, are widely in regulating apoptosis and cell survival, which can be activated by phosphorylation after a variety of different stresses and inflammation and cause excessive generation of MAPK-regulated genes, uncontrolled proliferation and cell death. According to our western blot data, p-JNK and p-p38 expressions were obviously decreased, while total JNK and total p38 expressions were not changed significantly after treatment with CA in *rd10* mice (Fig. 5b,c). It may be related to the amelioration of oxidative stress by CA treatment. In order to further reveal this effect in different layers of retina, p-JNK was tested by immunohistochemistry (Fig. 5a). Our images show that there was strong expression of p-JNK in ONL and the inner segment (IS) of the vehicle group, which was significantly ameliorated after CA treatment (Fig. 5a–c). Our data further confirms that CA plays its role of antioxidant in the photoreceptors of *rd10* mice.

#### Down regulation of expression of GRP78, ATF4, ATF6 and p-IRE1α

**Figure 6:**
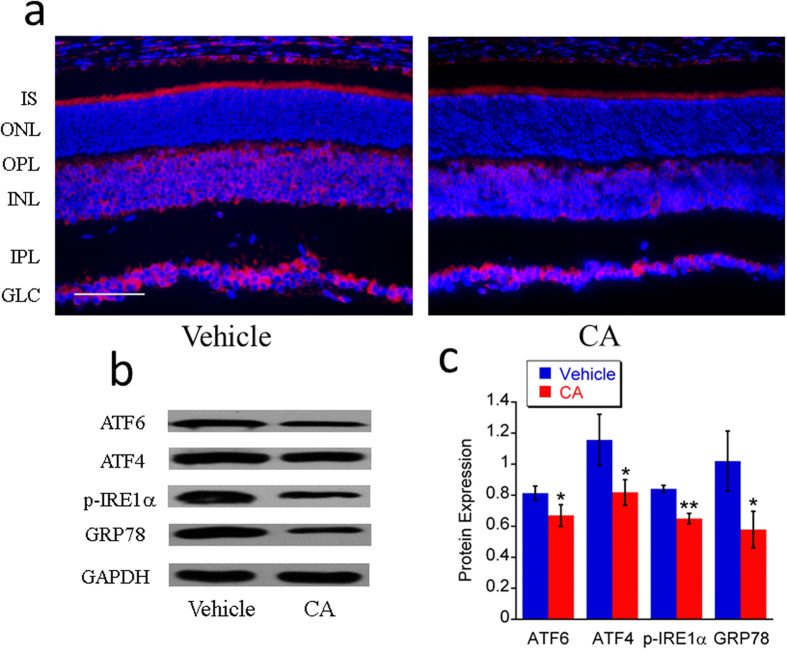
CA treatment downregulated the expression of some markers of ER stress in *rd10* retina at P21. CA treatment inhibits the expression of some markers of ER stress. **a**. Representative images (20x) of retinal immunohistochemistry for GRP78/BiP (red). Scale bar indicates 50 μm. **b**. Representative images of western blot for GRP78/BiP, p-IRE1α, ATF4 and ATF6. **c**. Average protein expression of GRP78/BiP, p-IRE1α, ATF4 and ATF6 tested by western blot. *n* = 3 in each group. The error bars indicate standard deviations. *p < 0.05, **p < 0.01 *vs*. vehicle group.

ER stress may result in cell death which causes photoreceptor degeneration. Four protein markers of ER stress (GRP78, ATF4, ATF6 and p-IRE1α) were detected in this study to understand whether CA treatment can reduce ER stress in *rd10* retina. Western blot was performed to detect the change of these proteins after the CA treatment. Our data shows that all these proteins decreased significantly after treatment with CA in *rd10* mice compared to the vehicle group (Fig. 6b). Because GRP78 is a master regulator of the unfolded protein response in ER, immunohistochemistry was performed to detect GRP78 *in situ* (Fig. 6a). Our images reveal that GRP78 exists in inner segment, inner nuclear layer, and ganglion cell layer, which is consistent with the finding of Nookala *et al.*^39^ and our previous finding^40^. After CA treatment, GRP78 was mainly downregulated in the inner segment of photoreceptors and inner nuclear layer (Fig. 6a,b), which implies that ER stress is reduced after CA treatment.

#### Upregulation of SIRT1 expression and down-regulation of p-p65 expression

**Figure 7:**
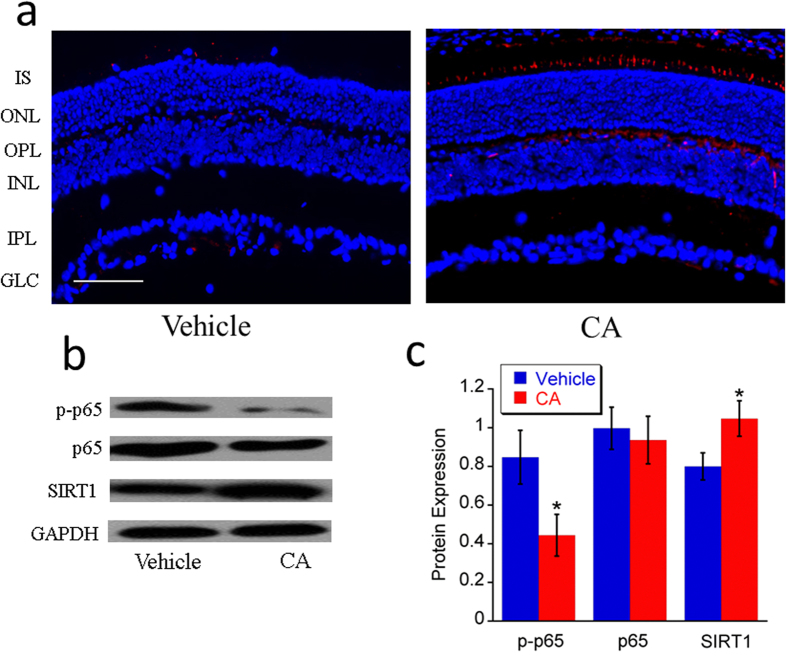
CA treatment upregulated SIRT1 expression and downregulated p-p65 expression in *rd10* retina at P21. CA treatment upregulated SIRT1 expression and downregulated p-p65 expression. **a**. Representative images (20x) of retinal immunohistochemistry for SIRT1 (red). Scale bar indicates 50 μm. **b**. Representative images of western blot for SIRT1, p65 and p-p65. **c**. Average protein expression of SIRT1, p65 and p-p65 tested by western blot. *n* = 3 in each group. The error bars indicate standard deviations. *p < 0.05 *vs*. vehicle group.

Sirtuin type 1 (SIRT1) is a member of mammalian sirtuin family that generates enzyme activity in a NAD^+^-dependent way to deacetylase histones and prolong survival. In this study, the expression of SIRT1 increased after treatment with CA (Fig. 7b,c). Moreover, SIRT1 was mainly upregulated in the photoreceptor inner segment and outer plexiform layer in CA-treated retina of *rd10* mice (Fig. 7a). It may provide neuroprotective effect to the photoreceptors.

NF-κB (nuclear factor-kappaB) is a heterodimeric protein consisting of p50, p52, p65, RelB, and Rel and is related to inflammatory reaction. The presence of inflammatory reaction was observed in the eyes of *rd10* mice and patients with RP. The chronic inflammation may play a pathogenic role in RP^41^,^42^. Therefore, p-p65 was tested with western blot to study relationship of CA treatment and NF-κB pathway. Our data shows that CA treatment could inhibit the activation of p-p65 in *rd10* mice (Fig. 7b,c), which means CA can control the inflammatory responses.

## Discussion

RP is a type of hereditary retinal diseases with progressive degeneration of photoreceptors characterized, as the result of rod-specific gene mutations. About a hundred genes are known to be related to RP^43^,^44^. More than 50 genes are found to be relevant to non-syndromic RP and about 3100 mutations have been revealed in these genes. Mutations in 29 genes are found to cause syndromic RP, in which 12 genes are associated with Usher syndrome and 17 genes cause Bardet-Biedl syndrome. There are 1200 mutations in these 29 genes. Despite the genetic heterogeneity, RP seems to involve elevated oxidative stress and ER stress and the photoreceptors seem to die by a common apoptotic mechanism^6^,^14^,^45^,^46^. Retina is particularly sensitive to oxidative damage because of its high oxygen demand and high content of unsaturated lipids^47^. The redox balance is disrupted in RP, which is associated with photoreceptor apoptosis and degeneration^6^. Due to the imbalance between the intracellular oxidative and anti-oxidative defense system, supplement of external antioxidants is needed to eliminate excessive oxidative stress as a potential therapeutics in RP. This raises the idea that these processes could be impacted pharmacologically and therefore be applicable to many genetic forms of RP. This idea has been supported by other studies that demonstrated reducing oxidative stress slowed the rate of photoreceptor degeneration^11^,^17^,^18^,^20^,^48^. Here we have demonstrated that CA administration had a similar neuroprotective effects against photoreceptor death in the *rd10* mouse model of RP. Our data showed that CA improved photoreceptor function, reduced photoreceptor cell death and degeneration. In addition, our study reveal the protein pathways of oxidative stress, ER stress and inflammatory response affected by CA treatment, which are the possible mechanisms underlie CA protective effect in *rd10* retinas.

A lot of evidences have shown oxidative stress is one of the main pathogenic factors contributing to photoreceptor cell death in RP, which is probably related to the activation of Nrf2 pathway, MAPK pathway and NF-κB pathway^48^,^49^. Our data show that JNK and p38 subgroups of MAPKs, as well as NF-κB p65 were downregulated significantly in CA treated group than in the vehicle group, which implies that these pathways may be related to the photoreceptor degeneration in *rd10* mice and CA takes effect through these cellular pathways. Previous study reported that p38 MAPK can activate NF-κB pathway^50^. Further study is required to clarify the role of MAPKs and NF-κB pathway in the retinal cell death.

Our data shows ER stress was reduced by CA treatment in *rd10* mice according to the downregulation of GRP78, ATF4, ATF6 and p-IRE1α. Protein misfolding in the ER leads to the pathogenesis of many diseases. Activation of the unfolded protein response (UPR) causes oxidative stress and induces apoptosis through generating reactive oxygen species^51^. Chemical intervention, such as antioxidant, to reduce reactive oxygen species could improve protein folding and cell survival, providing an effective route to treat diseases caused by ER stress^52^,^53^. ER stress has been established as a pathogenic factor contributing to photoreceptor cell death^54^,^55^,^56^. Pathological ER stress will activate the apoptotic process leading to cell death and degeneration, and chronic ER stress has been shown to promote inflammatory response through activation NF-κB^57^,^58^. In addition, ER stress can also activate MAPKs pathway to induce autophagy^59^,^60^,^61^, and trigger apoptosis^62^. Therefore, CA treatment in *rd10* mice may play its role of neuroprotection partially through the inhibition of ER stress. It was reported that ER stress can be reduced by antioxidants^51^ through the elimination of reactive oxygen species, and oxidative stress and ER stress are closely linked together^63^. Further study is necessary to understand the underlying mechanism of CA in regulation of ER stress.

SIRT1 was reported to be pivotal in the regulation of cell fate in the response of cellular stress in mammalian cells and protect cells from death caused by oxidative stress^64^. Inhibiting SIRT1 by pharmacological inhibitor or SIRT1 siRNA significantly promotes apoptotic neuron death^65^. The deficiency of SIRT1 also causes retinal damage. In SIRT1-deficient mice, multiple retinal cell layers were significantly thinner than in normal eyes and the ONL was disorganized^66^. In *rd10* mice, a previous study showed that SIRT1 expression is strong at P15 and gradually decreases after that age in ONL^67^. Our data of SIRT1 immunostaining in *rd10* mice of vehicle group is similar to their data at P24 while the *rd10* CA-treated group showed upregulation of SIRT1 in the inner segment layer and outer plexiform layer. Numerous protein targets can be deacetylased by SIRT1, thereby regulating multiple cellular pathways related to stress responses, apoptosis and inflammation^68^. SIRT1 deacetylates the DNA repair factor Ku70 to reduce the disruption of Ku70-Bax interaction, keeping Bax away from mitochondria, thereby inhibiting apoptosis^69^. It was also reported that SIRT1 releases ER stress to protect cell against dysfunction^70^. Moreover, SIRT1 played its antioxidant role through the FOXO family^71^ and down-regulated the pro-inflammatory factor NF-κB directly by deacetylating the p65 subunit of NF-κB complex^72^. In the present study, SIRT1 was upregulated after treatment with CA in *rd10* mice, followed by the inhibition of pro-inflammatory factor NF-κB. Reactive oxygen species is a possible factor which links oxidative stress to SIRT1 pathway as a SIRT1 inhibitor^73^. CA may reduce the level of reactive oxygen species so that SIRT1 can be upregulated.

Apoptosis was considered as the main pathway of cell death in hereditary retinal degeneration in previous studies^74^,^75^. However, recent reports revealed the involvement of alternative cell death pathways in neuronal degeneration, featured by the activation of histone deacetylase, poly-ADP-ribose-polymerase (PARP), calpain, as well as accumulation of cyclic guanosine monophosphate and poly-ADP-ribose, and calcium overload^76^,^77^,^78^. In this alternative pathway, the activities of calpain and PARP co-localize to a large extent with the TUNEL assay^79^,^80^. In our study, the photoreceptor cell death may include apoptotic and non-apoptotic death^33^ and all kinds of dying cells^30^. Further study is necessary to explore the exact death mechanism of photoreceptor cells in *rd10* mice.

In summary, our results imply that CA plays the role of anti-oxidation, anti-ER stress and anti-inflammation through the regulation of multiple cellular pathways in *rd10* mice. For further studies of CA in the control of RP, more RP models with different mutations in different genes can be studied. The combination of CA with other antioxidants can also be explored. While genetic based treatments are developed, this type of anti-stress treatment may be used to slow RP progression with long-term administration. Further study is necessary to observe the long-term effect and toxicity of CA and decide whether multiple antioxidants should be used alternatively to reduce side-effects and extend the treatment effect for longer period. Our study provides scientific rationale for further study of CA as a potential supplementary treatment of RP in the future.

## Materials and Methods

### Animals and treatment

All animal procedures were approved by the Institutional Animal Care and Use Committee of the Cleveland Clinic Foundation and were conducted in accordance with the regulation of the ARVO Statement for the Use of Animals in Ophthalmic and Vision Research. The breeding pairs of C57BL/6J (WT mouse, Stock Number: 000664) and *rd10* mice (Stock Number: 004297) were purchased from the Jackson Laboratory (Bar Harbor, Maine). Carnosic acid (CA, 15 mg/kg, Enzo Life Sciences, Farmingdale, NY) dissolved in canola oil (0.91 mg/mil, Sigma-Aldrich, St. Louis, MO) or equal volume of canola oil were administered by intraperitoneal injection to *rd10* mice daily from P6 to P20 or P27 because *rd10* mice were reported to start retinal abnormality from P7^81^. At P21 or P28, a series of electroretinograms (ERGs) were recorded. Eyes were then harvested for anatomical and protein studies.

### ERG

After overnight dark adaptation, mice were anesthetized with a mixture of ketamine (80 mg/kg) and xylazine (16 mg/kg) diluted in saline. The pupils were dilated with eye drops (2.5% phenylephrine HCl, 1% cyclopentolate HCl, 1% mydriacyl) and the corneal surface was anesthetized with 0.5% proparacaine HCl eye drops. Mice were placed on a temperature-regulated heating pad during the ERG recording session. The tester was double-blinded to the treating status of the mice during the ERG test. To analyze the retinal function, we used the published protocol^40^,^82^,^83^ for dark-adapted and light adapted ERG tests. In dark-adapted session, the flash luminance ranged from 3.6 to 2.1 log cd s/m^2^. In light-adapted session, the flash luminance ranged from −0.8 to 1.9 log cd s/m^2^. The time for light-adaptation was 7 minutes before the first light-adapted ERG was recorded.

### Preparation of frozen sections

Mouse eyes were enucleated, fixed in 4% paraformaldehyde for 20 min at 4 °C, punched a hole at corneal limbus for prevention of the shrinkage of the eyeball and further fixed in 4% paraformaldehyde for 4 h at 4 °C. Eyes were then processed through a graded series of sucrose in phosphate-buffered saline (PBS) solutions (10% for 1 h, 20% for 1 h and 30% for overnight) at 4 °C, and embedded in optimum temperature cutting compound. Samples were frozen on dry ice and stored at −80 °C. Frozen sections (10 μm) were cut sagittally passing through the optic nerve head and placed onto slides.

### TUNEL

Apoptosis and necrosis was detected using the *In Situ* Cell Death Detection Kit (Roche Applied Science, Indianapolis, IN). 10 μm retinal sections were prepared as described above and incubated with freshly prepared 0.1% Triton X-100/0.1% sodium citrate permeabilization solution for 2 min on ice. After rinsing with PBS 3 times, sections were incubated with the TUNEL reaction mixture for 60 min at 37 °C in the dark and then rinsed with PBS 3 times. Sections were mounted with VECTASHIELD mounting medium with 4′,6-diamidino-2-phenylindole (DAPI) (Burlingame, CA), and visualized with the fluorescence microscope.

### Histology

After treatment, intact mouse eyes were enucleated, fixed in Karnovsky’s fixative (2% paraformaldehyde and 2.5% glutaraldehyde in 0.1 M phosphate buffer) for 60 min, removed the cornea and further fixed in Karnovsky’s fixative overnight. Then the eyes were fixed in 1% osmium tetroxide, dehydrated in graded ethanol and then propylene oxide. Next, eyes were transferred to a plastic resin mixture containing Polybed 812 and Araldite 502 (Polysciences) with polymerizer. After polymerization, five to ten sections of 1 μm-thickness were sectioned sagittally, passing through the optic nerve head. Slides were placed 1–2 drops of 1% toluidine blue in 1% sodium borate on a hot plate for 20 s and then rinsed with a gentle stream of distilled water to wash off the excess stain. Slides were covered by coverslips and visualized under the microscope. Pictures were taken on either side of the optic nerve head using a microscope, and the thickness of the ONL and INL was measured at 200 μm from the edge of the optic disc using ImageJ 1.48v software (National Institutes of Health, MD).

### Immunohistochemistry

Protein expression and location was examined by *in situ* immunofluorescence staining. Sections were incubated in PBS containing 5% normal goat serum, 1% bovine serum albumin (BSA) and 0.5% Triton X-100 for 1 h to block non-specific binding, followed by incubation with primary antibodies (anti-GRP78/BiP, 1:400, ab21685; anti-p-JNK, 1:100, ab124956; anti-SIRT1, 1:50, ab12193, Abcam, Cambrigde, MA) overnight at 4 °C. After three washes with PBS, sections were incubated with goat anti-rabbit IgG H&L Alexa Fluo^®^ 555 (1:600, ab150086, Abcam, Cambrigde, MA) for 2 h at room temperature. After washing with PBS, sections were mounted with VECTASHIELD mounting medium with DAPI (VECTOR LABORATORIES, Burlingame, CA) and examined under a fluorescent microscope.

### Western blot

Total cellular and nuclear protein was extracted from retinas and quantified using bicinchoninic acid assay kit. Homogenate in 2 × sodium dodecyl sulfate (SDS) sample buffer was boiled for 5 min, and then equal amounts of protein (40 μg) from each sample were subjected to electrophoresis on a 10% (v/v) SDS-polyacrylamide gel. After proteins were electroblotted to a polyvinylidene difluoride membrane, the membrane was blocked with Phosphate-buffered saline containing 5% dried non-fat milk or 3% BSA at room temperature for 1 h, and incubated with indicated primary antibodies (anti-ATF4, 1:1000, SC-200, Santa Cruz Biotechnology, Dallas, TX; anti-ATF6, 1:1500, ab37149; anti-GRP78/BiP, 1:2000, ab21685; anti-pIRE1α, 1:1000, ab48187, Abcam, Cambrigde, MA; anti-Nrf2, 1:1000, SC-722; Santa Cruz Biotechnology, Dallas, TX; anti-p38, 1:500, ab27986; anti-p-p38, 1:1000, ab4822; anti-JNK, 1:1000, ab59227; anti-p-JNK, 1:2000, ab124956; anti-p65, 1:600, ab7970; anti-p-p65, 1:2000, ab86299; anti-SIRT1, 1:2000, ab12193, Abcam, Cambrigde, MA) at 4 °C overnight, followed by incubating with the goat-anti-rabbit horseradish peroxidase-conjugated secondary antibody for 2 h. After incubation, membrane was washed three times, and the antigen-antibody complexes were visualized by the enhanced chemiluminescence system (PerkinElmer, Akron, OH).

### Statistical analysis

For the analysis of ERG data, two-way repeated measure ANOVA was used. The power analysis was conducted by the F-test of one-way ANOVA, where we considered numbers as outcome and groups as the factor. All other comparisons were made by one-way ANOVA. *p* < 0.05 was considered significant.

## Additional Information

**How to cite this article**: Kang, K. *et al.* Carnosic acid slows photoreceptor degeneration in the *Pde6b^rd10^* mouse model of retinitis pigmentosa. *Sci. Rep.* **6**, 22632; doi: 10.1038/srep22632 (2016).
